# The use of a predictive statistical model to make a virtual control arm for a clinical trial

**DOI:** 10.1371/journal.pone.0221336

**Published:** 2019-09-04

**Authors:** Jeffrey M. Switchenko, Arielle L. Heeke, Tony C. Pan, William L. Read

**Affiliations:** 1 Department of Biostatistics & Bioinformatics, Rollins School of Public Health, Emory University, Atlanta, GA, United States of America; 2 Levine Cancer Institute, Atrium Health, Charlotte, NC, United States of America; 3 Institute for Data Engineering and Science, Georgia Institute of Technology, Atlanta, GA, United States of America; 4 Department of Hematology and Medical Oncology, Winship Cancer Center of Emory University, Atlanta, GA, United States of America; Harran Universitesi, TURKEY

## Abstract

**Background:**

Randomized clinical trials compare participants receiving an experimental intervention to participants receiving standard of care (SOC). If one could predict the outcome for participants receiving SOC, a trial could be designed where all participants received the experimental intervention, with the observed outcome of the experimental group compared to the prediction for those individuals.

**Methods:**

We used the CancerMath calculator to predict outcomes for participants in two large clinical trials of adjuvant chemotherapy for breast cancer: NSABPB15 and CALGB9344. NSABPB15 was the training set, and we used the modified algorithm to predict outcomes for two groups from CALGB9344: one which received standard of care (SOC) chemotherapy and one which received paclitaxel in addition. We made a prediction for each individual CALGB9344 participant, assuming each received only SOC.

**Results:**

The predicted outcome for the group which received only SOC matched what was observed in the CALGB9344 trial. In contrast, the predicted outcome for the group also receiving paclitaxel was significantly worse than what was observed for this group. This matches the conclusion of CALGB9344 that adding paclitaxel to SOC improves survival.

**Conclusion:**

This project proves that a statistical model can predict the outcome of clinical trial participants treated with SOC. In some circumstances, a predictive model could be used instead of a control arm, allowing all participants to receive experimental treatment. Predictive models for cancer and other diseases could be constructed using the vast amount of outcomes data available to the federal government, and made available for public use.

## Introduction

In oncology, there exist statistical models which predict the likelihood of various outcomes for an individual with a given cancer diagnosis. The underlying formulas used by CancerMath and similar web-based programs add the risk of death conferred by disease and treatment related variables to the underlying actuarial risk of death to generate predicted outcomes.[1, 2] As described on CancerMath.net

*The goal of Cancer-Math.net is to provide medical professionals with web-based calculators for accurately predicting the clinical outcome for individual cancer patients*, *as well as for accurately estimating the impact of various treatment choices on that outcome*.

Programs like CancerMath can help an individual or her physician make clinical decisions. For an individual, it is no great surprise when a predicted outcome with a 25% chance of occurring actually takes place. In a group of 1000, an event with a 25% chance of occurring will probably happen to 250 individuals, with numbers higher or lower than 250 increasingly improbable. Statistical predictions of outcomes for large groups are more reliable than predictions for individuals because chance events balance out as numbers increase. This prediction of likely outcomes for a group is the goal of actuarial science, based on techniques developed before allopathic medicine was recognizable as such. For centuries, actuaries have used predictive statistical models to guide the pricing of insurance policies and other financial endeavors, the profitability of which depend on correct estimation of risk.

Predictive statistical models are not used in place of control arms in phase III clinical trials. If the control and intervention arms are truly equivalent, then the design of such a trial requires no knowledge of the natural history of the disease or of variables that were important in the past. All other variables cancel out and the only important variable is that of the intervention. To apply the result of a phase III trial in clinical practice–for example the finding that adjuvant chemotherapy improves survival for breast cancer–one must assume that conditions at the time the trial was conducted are similar enough to conditions in the present that the results of the trial remain relevant. Additionally, the variables affecting outcome in the trial need to remain relatively constant for the clinical trial to be useful.

If the range of variables remains similar in new patients to what was previously observed, the model could interpolate to predict new patient outcomes in a clinical trial participant who receives established standard-of-care treatment. The predictions for each participant would then be combined into a prediction for the group as a whole. This group prediction or “virtual control” arm could be used instead of an actual control group of participants in a phase III clinical trial who demonstrate the outcome of standard-of-care treatment by receiving it in real life. In a phase III clinical trial with a virtual control arm, all participants would receive experimental treatment. The outcome of the study would be determined by how much, if at all, the observed outcome of those participants diverge from the predicted outcome for that group of individuals if they had received standard of care. With this approach, safety signals from the experimental arm would be analyzed against historical safety data for the virtual control arm receiving standard of care therapy.

It has previously been proposed that the predictive statistical models underlying cancer nomograms, or visual prediction tools for assessment of risk or determination of outcomes, could be used to augment clinical trials. Using a phase II study of prostate cancer as an example, Koziol proposed that one could use a nomogram to predict outcomes for a group of study participants in a phase II study, and so gauge how the observed outcomes for the study differ from the predicted outcomes.[3] Building on this premise Jia et al built a predictive model using a prostate cancer nomogram and used this to predict the outcome of 155 cases of prostate cancer.[4]

To determine whether a virtual control arm could be successfully used in a large clinical trial, we used the model underlying the CancerMath breast cancer model to predict patient outcomes from two data sets from mature clinical trials of adjuvant chemotherapy for breast cancer: Cancer and Leukemia Group B (CALGB) 9344 and National Surgical Adjuvant Breast and Bowel Project (NSABP) B15. CALGB 9344 was a phase III study which produced a practice changing result: the finding that for study participants with hormone-receptor negative breast cancers, the addition of paclitaxel (P) to adjuvant chemotherapy with doxorubicin and cyclophosphamide (AC) improved survival over that seen with AC alone.[5] NSABP B15 was a phase III study randomizing participants thought to have tamoxifen-nonresponsive breast cancer to adjuvant chemotherapy with either AC; cyclophosphamide, methotrexate and fluorouracil (CMF); or a combination of both.[6] For our study and analysis, NSABP B15 served as the training set to generate a multiplier to better predict survival at 15 years with the CancerMath algorithm, and CALGB 9344 then served as the validation set to determine if a reliable virtual control arm can be produced by statistical models.

Our hypotheses:

1a. Null hypothesis: Assuming treatment with AC alone and starting with the characteristics of individual CALGB 9344 participants who did receive AC alone, the predictive model will generate a virtual control arm with predicted yearly survival outcomes which is equal to the observed survival of the CALGB 9344 AC only group.
1b. Alternative hypothesis: Observed survival for the group receiving AC alone is not equal to predicted survival for this group, assuming AC alone.
2a. Null hypothesis: Assuming treatment with AC alone and starting with the characteristics of individual CALGB 9344 participants who received both AC + P, the predictive model will generate a virtual control arm with predicted yearly survival outcomes which is equal to the observed survival of the CALGB 9344 experimental group.
2b. Alternative hypothesis: Observed survival for the group receiving AC+ P is not equal to predicted survival for this group, assuming AC alone.

We expected to fail to reject the null hypothesis for #1, because of an accurate prediction by the model for participants receiving AC only. We expected to reject the null hypothesis for #2 because the addition of P improved outcomes over what the model predicted with standard of care (AC only).

## Methods

For our study and analysis, NSABP B15 served as the training set, and we developed a multiplier correction to the CancerMath predictive model such that given the starting characteristics of NSABP B15 participants, the predicted survival at 15 years matched the observed survival of the actual NSABP B15 trial participants at 15 years. We then partitioned the predicted mortality burden at 15 years across each preceding year using a yearly mortality fraction derived from the Surveillance, Epidemiology, and End Results (SEER) database.

### CALGB 9344: Validation set

We used the CancerMath model with the multiplier correction to predict survival at 15 years for the subset of CALGB 9344 participants with the same age range, stage and hormone-receptor status as the NSABP B15 participant categories used in the training set. For this subset of CALGB 9344 participants, we compared predicted versus observed mortality for two groups: those who received adjuvant AC + P and those who received only AC. We again distributed the 15-year predicted mortality burden over preceding years.

### Training data set: NSABP B15

After receiving approval from the Emory University Institutional Review Board, NRG Oncology provided the dataset for NSABP B15, stripped of personal identifiers. NSABP B15 was a large study of adjuvant chemotherapy for breast cancer, accruing between 1984 and 1988.[6] Participants were between 20 and 59 years old, had breast cancer with at least one positive node, and were thought to be tamoxifen-nonresponsive. Women younger than 49 regardless of receptor status were included, whereas women 49–59 years old were included if they had non-estrogen receptor (ER)+/progesterone receptor (PR)+ tumors. For the training set we excluded all participants with ER+/PR+ cancers to best evaluate the role of the addition of P, which was most efficacious in hormone receptor negative tumors in CALGB 9344. Study participants received AC, CMF or both. No difference was observed between these chemotherapy groups in regard to survival, and CancerMath treats these two chemotherapy regimens as equivalent with respect to survival benefit.

### Automation of CancerMath

The web-based nomogram calculators at http://cancer.lifemath.net allow interactive explorations of treatment effects on mortality risk given a patient's demographic and clinical information. Unlike other programs, the source code for CancerMath is available on-line, well documented, and free for scientific use.[7] In order to perform calculations for a multiple subject population, we adapted the CancerMath implementation for automated processing. We extracted the JavaScript of the original web-based nomogram calculators with permission from the author, and transformed the code automatically based on a set of predefined rules to expose the input and output variables. The code transformations are limited to function interfaces, thus the correctness of the algorithm implementation and the output are preserved. This minimal transformation also allows for easy upgrade to newer versions of nomogram implementations and adaptation of new nomogram calculators.

A wrapper script was used to invoke the transformed nomogram functions using subject data in a CSV file as input, perform post processing tasks such as interpolating the mortality risk at monthly intervals, and output the risk data to files for further analysis. The wrapper script leverages a user defined mapping to assign data elements in the input CSV files to the nomogram input variables. The transformed code as well as the wrapper script requires only Node.js as the runtime environment, and can be deployed on servers or desktop systems. The transformation code and the wrapper script, referred to as AutoCM, are available at https://bitbucket.org/tcpan/autocm under Apache 2.0 license. The CancerMath logic remains under its own license.

### Training set: Mortality prediction

Based on patient age, tumor diameter (cm), number of positive nodes, ER status, PR status, hormone therapy, and chemotherapy type, CancerMath predicts the chance of an individual surviving at 15 years. In the training data set, we first produced a database of virtual participants with variables corresponding to the actual participants in NSABP B15. Treatment for the virtual participants was set to the “first generation regimen” for all participants, which in CancerMath includes AC and CMF, and matched demographic and clinical features were entered to generate a predicted survival for each virtual participant. We averaged the predicted 15-year survival points for the virtual population and compared this to the observed Kaplan-Meier estimate of 15-year survival.

### Validation data set: CALGB 9344

After receiving approval from the Emory University Institutional Review Board, the Alliance for Clinical Trials in Oncology Foundation provided the dataset for CALGB 9344, stripped of personal identifiers. CALGB 9344 was a large study of adjuvant chemotherapy for breast cancer, accruing between 1994 and 1999. The dataset contained complete data for 2651 study participants, of whom 1378 received AC + P and 1273 received AC. In CALGB 9344, all participants with ER+ cancers also received adjuvant tamoxifen. As noted previously, we excluded participants with ER+/PR+ cancers from the data analysis of both training and validation sets and retained those with any other receptor combination. We also excluded participants older than 60, which were present in the CALGB 9344 set but not in the NSABP B15 set.

With these adjustments, we produced a database of virtual participants with variables corresponding to the actual participants in CALGB 9344. Treatment for the virtual participants was again set to the “first generation regimen” for all participants, both those who did get only AC and those who received paclitaxel in addition. In CALGB 9344, participants with ER+ or PR+ received tamoxifen and this data was also input. For each participant, her 15-year predicted survival estimate from CancerMath was multiplied by a subgroup specific correction, estimated from the NSABP B15 data. Subgroups included combinations of ER/PR status (ER-/PR- or ER+/PR- or ER-/PR+) and age group (20–39 or 40–59). Yearly estimates of survival (1-year, 2-year, etc.) were adjusted using SEER (details below). Next, for each patient, we took sequential random draws *X*_*i*_ from the Uniform(0,1) distribution, where *i* = 1 to 15 years. If *X*_*i*_ < probability of death in year *i* given the patient survived to year *i–*1, then that simulated patient dies, and the survival time is recorded. Else, the patient proceeded to year *i+*1, and the process was repeated. If the patient’s simulation survived to 15 years, then the survival time was censored at 15 years. Survival estimates for both the observed and simulated data were reported at yearly intervals for those receiving AC+P and those receiving AC, and standard errors were estimated using Greenwood’s formula[8]. Ninety-five percent confidence intervals were reported, and significance was assessed at the 0.05 level. Comparisons at yearly intervals between observed and predicted arms were made using two-sided two-sample Z-tests. Statistical analysis was performed using R 3.3.1 [9]Due to the exploratory nature of this analysis, we did not adjust our analysis for multiple comparisons.

## Results

### Training set: Multiplier correction factor

We found that the CancerMath prediction model underestimated mortality for the NSABP B15 participants. In the NSABP B15 database, a total of 1,450 participants were ER-/PR-, ER+/PR-, or ER-/PR+, and all were aged 20–59. The highest frequency age-ER-PR groupings were 40–59 ER-/PR- (n = 263), 40–59 ER+PR- (n = 167), and 20–39 ER-/PR- (n = 108) (Table 1). Observed survival at 15 years for NSABP B15 age-ER-PR subgroups ranged from 33.7% (20–39 ER+/PR-) to 52.9% (40–59 ER-/PR+). Predicted survival using CancerMath ranged from 57.1% (40–59 ER+/PR-) to 67.1% (20–39 ER-/PR+). In total, the observed 15-year survival for the 1,450 NSABP B15 participants without ER+/PR+ breast cancers was 47.7% versus the predicted survival of 61.6%, a difference of 13.9%. To improve prediction accuracy for the subsequent validation set we estimated a multiplier correction to add to the CancerMath prediction. For each subgroup the multiplier correction was the observed survival divided by the predicted survival. These corrections ranged from 0.54 to 0.809, and equaled 0.775 overall (Table 1).

**Table 1:** NSABP B15(learning set) observed and predicted 15 year survival estimates.

Category	N	Observed survival	Predicted survival	Correction (Obs/Pred)
All B15cases, except ER(+)/PR(+)	1450	0.477	0.616	0.775
20–39 ER(-)/PR(-)	250	0.527	0.652	0.808
20–39 ER(+)/PR(-)	70	0.337	0.625	0.54
20–39 ER(-)/PR(+)	61	0.508	0.671	0.755
40–59 ER(-)/PR(-)	634	0.488	0.604	0.809
40–59 ER(+)/PR(-)	295	0.413	0.571	0.723
40–59 ER(-)/PR(+)	140	0.529	0.669	0.791

NSABP B15 is the training set. Subsets are defined using combinations of ER-PR status (ER-/PR-, ER+PR-, ER+PR-) and age group (20–39, 40–59). Observed survival is estimated using the Kaplan-Meier method. Predicted survival at 15 years for each patient is estimated using the CancerMath calculator. Predictions within each subset combination were averaged across patients. Column 5 is the ratio of observed to predicted survival which is used as a multiplier correction for predictions made for individuals within each subset in the validation set (CALGB 9344).

Technical report 10 available at CancerMath.net notes that the CancerMath predictive model was prone to “under-predicting short-term lethality for very lethal and ER- cancers“[7]. It is not within the scope of this project to determine exactly why CancerMath underpredicted mortality for participants on this trial.

### Training set: Probability density function / mortality fraction

For the participants in NSABP B15, observed survival at each yearly interval following diagnosis was used to generate a survival curve for the group as a whole. CancerMath predicts the 15-year mortality and then generates a predicted survival curve using a probability density function, with the yearly mortality fraction up to 15 years derived from that seen in the entirety of the SEER breast cancer cohort from 1986 through 2003.[7] For the NSABP B15 population, the curve generated by the CancerMath program overestimated survival in the years immediately following diagnosis.

We suspected that this overestimation of survival was because a function based on the entire SEER database skews towards outcomes for ER+/PR+ cancers, which are more common than cancers with other receptor types. The risk of death in the years 2 through 5 after diagnosis is greater for non ER+/PR+ cancers as compared to ER+/PR+.[10] To confirm this, we used the SEER database to identify 92,701 patients with a primary breast cancer diagnosed between 1993 and 1996. The Kaplan-Meier survival estimate for overall survival at 15 years was 47.8%, and of the 52.2% mortality across the 15 years, 10.3% occurred in the 1^st^ year, 10.3% in the 2^nd^ year, 9.5% in the 3^rd^ year, 8.6% in the 4^th^ year, and 61.3% occurred in the remaining 11 years (Table 2). In the subset of this group who matched those in our training set (not ER+/PR+, aged 20–59 and Stage II/III) we identified 7,139 patients. The Kaplan-Meier estimate for overall survival at 15 years was 55.6%, and of the 44.4% mortality across the 15 years, 6.4% occurred in the 1^st^ year, 19.8% in the 2^nd^ year, 15.5% in the 3^rd^ year, 12.8% in the 4^th^ year, and 45.5% occurred in the remaining 11 years (Table 2). A revised mortality fraction was produced using the 7139 patients in this subset. Returning to the training set, this new mortality fraction was used to allocate a percentage of the predicted 15-year total mortality burden to each preceding year.

**Table 2:** Mortality fraction: Percentage of 15 year total mortality occurring each year among breast cancer cases in SEER 1993–1996.

Year	Number at Risk (All cases)	Percent died (All cases)	Number at Risk (non ER+/PR+ subset)	Percent died (non ER+/PR+ subset)
0	92701	0	7139	0
1	87974	0.103	6967	0.064
2	82952	0.103	6335	0.198
3	78211	0.095	5826	0.155
4	73998	0.086	5412	0.128
5	70255	0.077	5131	0.084
6	66912	0.067	4915	0.065
7	63765	0.064	4746	0.05
8	60773	0.06	4594	0.045
9	58027	0.056	4470	0.036
10	55293	0.054	4354	0.032
11	52635	0.053	4256	0.029
12	50179	0.049	4147	0.03
13	47771	0.047	4019	0.036
14	45558	0.044	3933	0.025
15	43344	0.042	3833	0.024

The mortality fraction was defined as the percent of total mortality at year 15 occurring each year through Year 15. The percent at Year X was estimated by dividing the total mortality estimate through Year 15 (1 minus KM at 15 years) by the difference in survival between Years X-1 and X. Columns 2 and 3 represent number at risk and mortality fraction for all SEER cases with a breast cancer primary site (C500-C509) from 1993–1996. Columns 4 and 5 represent the number at risk and mortality fraction for the following subset: ER(-)/PR(-) or ER(+)/PR(-) or ER(-)/PR(+), ages 20–60, Stage II/III

### Validation set

With the new yearly mortality fraction from SEER using only stage 2 and 3, non ER+/PR+ cases, and the new multiplier correction for the 15-year mortality prediction, the training stage of the project was complete and we began the validation stage. In the CALGB 9344 database, 679 patients aged 20–59 who were ER-/PR-, ER+/PR-, ER-/PR+ were assigned AC + P, and 685 were assigned AC. The 15-year observed Kaplan-Meier survival estimates for each subgroup, and the 15-year prediction survival estimates for each subgroup based on the CancerMath algorithm are displayed in Table 3. Looking at the ER-/PR- subgroups, for example, 15-year survival in experimental arm participants within the predicted virtual group treated with AC aged 20–39 years compared to the observed group treated with AC + P was 50.4% versus 64.9%, and 46.2% versus 52.5% in the group aged 40–59 years. Multiplier corrections from Table 1 were multiplied by the predicted survival in each subgroup to obtain the adjusted predicted survival for each group. In the control comparisons (observed participants treated with AC alone compared to virtual participants treated with AC alone), 15-year survival rates were similar. Again looking closer at our ER-/PR- subgroup, 15-year survival within the predicted virtual group treated with AC aged 20–39 compared to the observed group treated with AC was 49.5% versus 49.7%, and 45.6% versus 45.4% in the group aged 40–59 years.

**Table 3:** CALGB 9344 (validation set) observed and predicted 15 year survival estimates.

Category	Treatment	N	Observed survival	Predicted survival (CancerMath)	Multiplier correction	Predicted survival (Final)
20–39 ER(-)/PR(-)	AC+P	121	0.649	0.624	0.8078	0.504
20–39 ER(+)/PR(-)	AC+P	23	0.549	0.714	0.5399	0.385
20–39 ER(-)/PR(+)	AC+P	27	0.536	0.631	0.755	0.476
40–59 ER(-)/PR(-)	AC+P	323	0.525	0.572	0.8085	0.462
40–59 ER(+)/PR(-)	AC+P	112	0.485	0.648	0.723	0.468
40–49 ER(-)/PR(+)	AC+P	73	0.583	0.614	0.7914	0.486
20–39 ER(-)/PR(-)	AC	145	0.497	0.613	0.8078	0.495
20–39 ER(+)/PR(-)	AC	26	0.182	0.722	0.5399	0.39
20–39 ER(-)/PR(+)	AC	29	0.572	0.646	0.755	0.488
40–59 ER(-)/PR(-)	AC	312	0.454	0.564	0.8085	0.456
40–59 ER(+)/PR(-)	AC	94	0.472	0.659	0.723	0.476
40–59 ER(-)/PR(+)	AC	79	0.625	0.6	0.7914	0.475

CALGB9344 is the validation set. Subsets are defined using combinations of ER-PR status (ER-/PR-, ER+PR-, ER+PR-) and age group (20–39, 40–59) used for learning set. Observed survival is estimated using the Kaplan-Meier method. Predicted survival at 15 years for each patient is estimated using the CancerMath calculator. Predictions were averaged across patients within each subset. Column 6 is the correction factor which is used as a multiplier for these subset combinations within CALGB. Column 7 is the final predicted survival after applying the multiplier.

**Fig 1:**
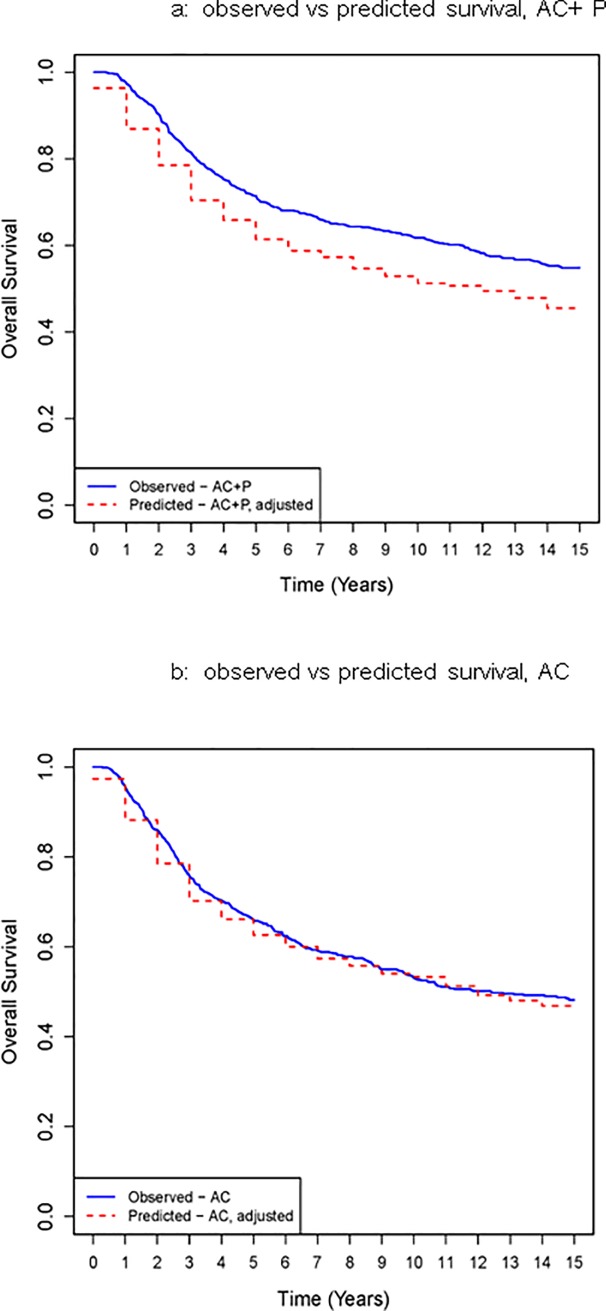
Predicted vs observed overall survival of CALGB patients receiving either AC+P (Fig 1A) or AC (Fig 1B).

Finally, overall observed survival for the AC + P and AC groups was estimated using the Kaplan-Meier method at yearly intervals and is shown in Tables 4 and 5. The NSABP corrections from Table 3 were multiplied by each patient’s 15-year survival estimate from CancerMath to obtain 15-year adjusted predicted survival estimates (Tables 4 and 5). The mortality fraction was then applied to obtain yearly estimates for predicted overall survival from Years 1 to 14 and random draws from the Uniform(0,1) distribution were utilized to simulate a predicted survival time for each patient. Observed and predicted yearly survival were compared, as shown in Fig 1.

**Table 4:** Survival estimates by year for CALGB 9344 subsets, observed and predicted–AC+P.

Time	N	Observed survival (95% CI)	Predicted, Adjusted (95% CI)	p-value
1	662	0.975 (0.963–0.987)	0.963 (0.949–0.977)	0.193
2	611	0.903 (0.881–0.925)	0.869 (0.844–0.894)	0.046
3	550	0.815 (0.786–0.844)	0.785 (0.754–0.816)	0.171
4	500	0.752 (0.719–0.785)	0.704 (0.669–0.739)	0.053
5	472	0.714 (0.681–0.747)	0.658 (0.623–0.693)	0.024
6	438	0.681 (0.646–0.716)	0.614 (0.577–0.651)	0.01
7	410	0.66 (0.625–0.695)	0.588 (0.551–0.625)	0.006
8	389	0.644 (0.607–0.681)	0.573 (0.536–0.61)	0.008
9	368	0.633 (0.596–0.67)	0.546 (0.509–0.583)	0.001
10	353	0.618 (0.581–0.655)	0.529 (0.492–0.566)	<0.001
11	339	0.602 (0.565–0.639)	0.513 (0.476–0.55)	<0.001
12	323	0.582 (0.543–0.621)	0.507 (0.47–0.544)	0.007
13	299	0.567 (0.528–0.606)	0.495 (0.458–0.532)	0.009
14	269	0.555 (0.516–0.594)	0.479 (0.442–0.516)	0.006
15	242	0.548 (0.509–0.587)	0.455 (0.418–0.492)	<0.001

Columns 2 represents the number at risk for AC+P at yearly intervals. Columns 3 represents the Kaplan-Meier estimates for overall survival at each year, along with 95% confidence intervals, for the observed data. Columns 4 represents the predicted survival estimates for overall survival at each year, along with 95% confidence intervals. Columns 5 represents p-values obtained by comparing the observed survival to the predicted survival using two-sided two-sample Z-tests.

**Table 5:** Survival estimates by year for CALGB 9344 subsets, observed and predicted–AC.

Time	N	Observed survival (95% CI)	Predicted, Adjusted (95% CI)	p-value
1	653	0.955 (0.939–0.971)	0.974 (0.962–0.986)	0.057
2	583	0.859 (0.834–0.884)	0.882 (0.858–0.906)	0.194
3	508	0.759 (0.728–0.79)	0.785 (0.754–0.816)	0.251
4	463	0.704 (0.669–0.739)	0.702 (0.669–0.735)	0.936
5	425	0.66 (0.625–0.695)	0.661 (0.626–0.696)	0.969
6	393	0.624 (0.587–0.661)	0.626 (0.591–0.661)	0.939
7	361	0.592 (0.555–0.629)	0.6 (0.563–0.637)	0.766
8	347	0.578 (0.541–0.615)	0.574 (0.537–0.611)	0.882
9	323	0.55 (0.511–0.589)	0.558 (0.521–0.595)	0.772
10	306	0.53 (0.491–0.569)	0.54 (0.503–0.577)	0.717
11	286	0.511 (0.472–0.55)	0.533 (0.496–0.57)	0.425
12	271	0.502 (0.463–0.541)	0.512 (0.475–0.549)	0.717
13	255	0.496 (0.457–0.535)	0.492 (0.455–0.529)	0.885
14	244	0.492 (0.453–0.531)	0.48 (0.443–0.517)	0.664
15	169	0.482 (0.443–0.521)	0.469 (0.432–0.506)	0.637

Columns 2 represents the number at risk for AC at yearly intervals. Columns 3 represents the Kaplan-Meier estimates for overall survival at each year, along with 95% confidence intervals, for the observed data. Columns 4 represents the predicted survival estimates for overall survival at each year, along with 95% confidence intervals. Columns 5 represents p-values obtained by comparing the observed survival to the predicted survival using two-sided two-sample Z-tests.

Plotted in Fig 1 is observed survival for study participants (aged 20–59, who were ER(-)ER(-)/PR(-), ER(+)/PR(-), ER(-)/PR(+)) as estimated by the Kaplan-Meier method, as well as simulated predicted annual survival rates for each arm, after applying the B15 multiplier correction and the SEER mortality fraction. A: population receiving AC+P. B: population receiving AC only.

Starting with Year 5, each yearly survival estimate for AC+P (Fig 1A) was statistically significantly different than the predicted value (p<0.05)–fitting with the published results of CALGB 9344 noting a statistically significant survival advantage with the addition of paclitaxel in hormone receptor negative breast cancers. Looking closer, at year 5, predicted and observed survival with AC + P were 65.8% and 71.4% (p = 0.024), respectively; and at years 10 and 15 were 52.9% and 61.8% (p<0.001), and 45.5% and 54.8% (p<0.001), respectively. In contrast, we failed to reject the null hypothesis that yearly observed estimates and predicted values for AC (Fig 1B) were significantly different (p>0.05).

## Discussion

After refining the CancerMath predictive model with processing of one training set (NSABP B15) and a receptor-subtype specific yearly mortality increment derived from SEER data, we were able to accurately predict the yearly mortality for a virtual control group within the validation set (CALGB 9344) receiving standard of care. We successfully showed that the group receiving experimental treatment (paclitaxel) along with standard of care (AC) outperformed the survival prediction made for the virtual control group predicated on treatment being only standard of care. Underscored further, we were able to reach a conclusion similar to that reached in the actual study about the benefits of paclitaxel without the need to enroll thousands of control patients to receive standard of care AC alone. We propose that with the use of a validated statistical predictive model, a clinical trial could evaluate the benefit of adding a new therapy to standard of care in a study where all participants receive the new combination and none receive only standard of care. The predicted outcome for the group would serve as the control arm. Our successful effort offers proof of principle.

A virtual control group produced by a statistical predictive model is not a historical control, as the term is commonly used. The construction of a historical control group means matching an individual trial participant to a single historic individual considered to be similar to him or her. Reducing an individual to a set of variables and using those variables to predict her outcome is not the same. Empirically deriving the influence of a variable from multiple historic individuals sidesteps idiosyncracies of time, place and randomness. A prediction based on these variables will be more reliable than one based on perceived similarity to a different individual.

Current methods of clinical trial design are becoming untenable. Funds for the conduct of large clinical trials are increasingly limited, and there is great interest in rapid, small single arm studies aimed at “accelerated approval” of interventions. A virtual control arm would strengthen phase II or accelerated approval studies that currently have no control arm[3], while avoiding ethical issues associated with placebo controls.[11] Clinical trials designed using a virtual control arm would also be more economical, as the control group is now a mathematical prediction and fewer participants will be required to enroll to complete the study, Additionally, current studies anticipate their accrual needs using the observed outcomes of historical groups of people perceived to be similar to the population of interest–a historical control group. A historical control group is not the same thing as the proposed virtual control method. The virtual control group is made in real time using variables specific to actual individuals enrolled in the trial. Accurately predicting the likelihood of an outcome means accurately judging the number of participants in the experimental group needed to demonstrate an effect on that outcome. Alternatively, a future trial may choose to enroll patients in a trial with a high ratio of active to standard-of-care (e.g., 5:1 or 10:1). From that trial, one could determine how well the virtual control predictions match with the observed control, but without the cost of many control patients. For all of these important reasons, we envision a meaningful path to employ predictive models to generate virtual control groups.

In the introduction to their book *Applied Predictive Modeling*, Kuhn and Johnson note “our abilities to predict or make decisions are constrained by our present and past knowledge and are affected by factors that we have not considered. These realities are limits of any model, yet these realities should not prevent us from seeking to improve our process and build better models.”[12] Testing any predictive statistical model against other retrospective and prospective datasets as they become available will allow refinement of the variables affecting survival, inclusion of new variables as they are discovered, and creation of a true actuarial-type predictive model. Predictive models like CancerMath have been developed for cancer diagnoses, and could be devised for myocardial infarction, head trauma, or any other discrete event which influences a clinically important end point. Improved predictive statistical models for cancer and other diseases would not only improve the efficiency of clinical trials, but would also improve individual patient management as was the intent of the CancerMath creators.

Predictive statistical models require time and money to create and maintain. This has proven worthwhile for finance and insurance companies whose models make predictions that are then used to turn a profit. As it regards to health outcomes, a predictive model like CancerMath is useful in individual patient management, and could be useful in the design and analysis of clinical trials, but in neither case can the prediction itself be directly used to make money. Who then should pay for these models, and how do they get a return on their investment? We propose that these models should be made and maintained by the federal government[13], as it is the role of government to undertake projects which are not in themselves profitable but that promote the common interest. Besides the very useful SEER dataset, the federal government is also privy to large private clinical datasets from two overlapping sources: NIH-funded clinical trials (such as those we were allowed to use) and those submitted by industry to the FDA. Disease specific models could be readily produced by actuaries using these rich data sources, perhaps jumpstarting efforts to incorporate validated nomograms into clinical practice, the potential benefits of which we have shown with this study. As a public service these predictive models could be available to all, and having been created by an impartial party, generated results would be more credible when used to validate or justify new interventions aimed at improving outcomes.
